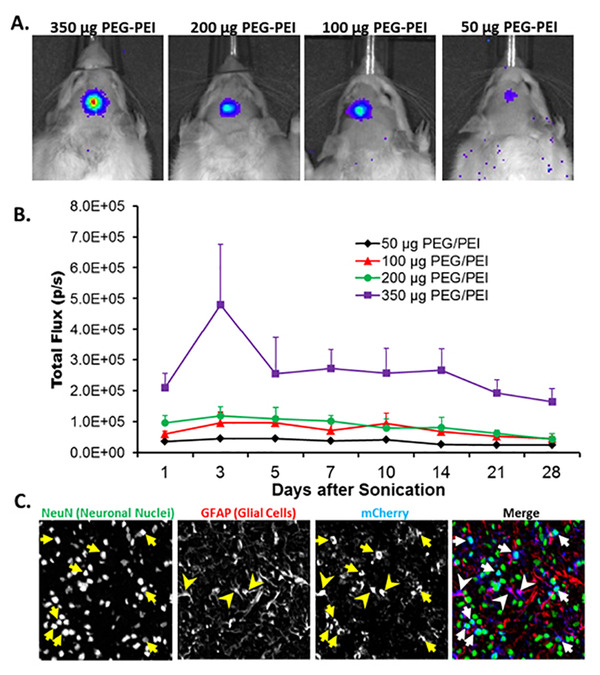# Ultrasound-targeted nanoparticle delivery across the blood-brain barrier

**DOI:** 10.1186/2050-5736-3-S1-O20

**Published:** 2015-06-30

**Authors:** Richard Price

**Affiliations:** 1 University of Virginia, Charlottesville, Virginia, United States

## Background/introduction

The delivery of systemically administered drugs and genes to the CNS is hindered by both the blood-brain barrier (BBB), which limits transport from the bloodstream to the brain to only a few privileged molecules, and the nanoporous electrostatically charged tissue space, denoted here as the “brain tissue barrier” (BTB). Our group engineers targeted drug and gene delivery approaches, capable of overcoming both of these physical barriers, for the treatment of brain tumors and neurodegenerative diseases. We focus on nanoparticle (NP) delivery systems, as they offer the potential for enhanced transfection efficiencies and controlled-drug release.

## Methods

To deliver drug- and gene-bearing NPs across the BBB, we use focused ultrasound (FUS) and contrast agent microbubbles (MBs). FUS may be applied using either MR-guidance or with a simple table-top system. We and others have shown that activating MBs with FUS yields safe and transient BBB opening in the FUS focal zone. Technologies for overcoming the BTB center on coating the drug and gene bearing NPs with an extremely dense brush layer of polyethylene glycol (PEG). NPs are injected at the time of BBB opening to permit their delivery to the CNS.

## Results and conclusions

**Figure 1:**
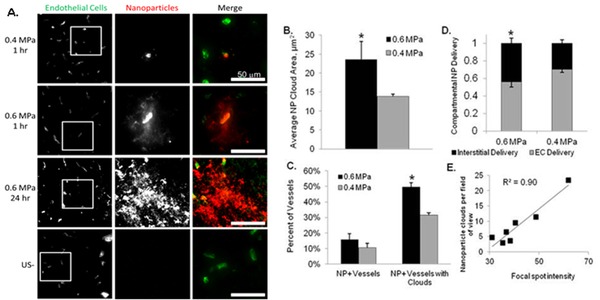


Drug Bearing NP Delivery – PEG-coated polystyrene (PS) tracer NPs (60 nm diameter) and biodegradable polylactide-co-glycolide (PLGA) NPs (75 nm) were engineered to penetrate brain tissue and then delivered across the BBB in rats using 1 MHz FUS. NPs were delivered to endothelium and as “clouds” to brain tissue. PS-NP delivery through the brain continued over 24 hours, yielding enhancements of cloud size and intensity (Figure 1A). FUS at 0.6 MPa delivered a larger fraction of PS-NP to the interstitial space (Figure 1D) and increased PS-NP coverage area (Figure 1B). The percentage of PS-NP+ vessels producing clouds was increased (P<0.05) to 50% at 0.6 MPa (Figure 1C). The higher US pressure produced a significant 4.6-fold increase in large (i.e. >200 μm2) PS-NP clouds. Focal spot intensity on MRI predicted the number of PS-NP clouds (Figure 1E). We also verified that BBB opening with MBs and FUS at 0.6 MPa can be used to substantially increase 60 nm PS-NP delivery to intracranial 9L rat tumors.

*Gene Bearing NP Delivery* — We used a blend of non-PEGylated and highly PEGylated polymers at an optimized ratio to engineer brain-penetrating DNA NPs with a polyethylenemine (PEI) core polymer. We delivered PEI-NPs (~50 nm in diameter) carrying either luciferase or mCherry plasmid DNA (driven by the unmethylated CpG-free β-actin promoter) across the BBB in rats using MR-guided FUS and MBs. Robust luciferase transgene expression, corresponding to a single focal site of FUS exposure, was visible (Figure 2A), and the intensity of gene expression was correlated with PEI-NP concentration (Figure 2B). After delivering mCherry PEI-NPs across the BBB with FUS and MBs, we immunochemically detected mCherry in both glial cells (GFAP, red) and neuronal cell nuclei (NeuN, green) (Figure 2C). mCherry expression was homogeneously distributed throughout the sonicated area (Figure 2C), demonstrating the benefit of combining FUS-mediated delivery across the BBB with brain-penetrating NPs. We believe these studies represent the first evidence for brain transfection via the delivery of a non-viral gene NP across the BBB with FUS. Going forward, this approach may be used to deliver genes for neurotrophic factors for the treatment of neurodegenerative diseases.

**Figure 2:**